# Research progress of N1-methyladenosine RNA modification in cancer

**DOI:** 10.1186/s12964-023-01401-z

**Published:** 2024-01-30

**Authors:** Yafeng Liu, Shujun Zhang, Xiaohui Gao, Yi Ru, Xinyu Gu, Xinjun Hu

**Affiliations:** 1 Department of Infectious Diseases, The First Affiliated Hospital, College of Clinical Medicine, Henan University of Science and Technology, Luoyang, No. 24 Jinghua Road, Jianxi District, 471000 Henan China; 2 Department of Oncology, The First Affiliated Hospital, College of Clinical Medicine, Henan University of Science and Technology, Luoyang, No. 24 Jinghua Road, Jianxi District, 471000 Henan China; 3 Hepatobiliary Pancreatic Surgery, The First Affiliated Hospital, College of Clinical Medicine, Henan University of Science and Technology, Luoyang, 471000 Henan China

**Keywords:** N1-methyladenosine, Detect method, Writers, Erasers, Readers, Regulation of cancer

## Abstract

N1-methyladenosine (m1A) is a post-transcriptionally modified RNA molecule that plays a pivotal role in the regulation of various biological functions and activities. Especially in cancer cell invasion, proliferation and cell cycle regulation. Over recent years, there has been a burgeoning interest in investigating the m1A modification of RNA. Most studies have focused on the regulation of m1A in cancer enrichment areas and different regions. This review provides a comprehensive overview of the methodologies employed for the detection of m1A modification. Furthermore, this review delves into the key players in m1A modification, known as the “writers,” “erasers,” and “readers.” m1A modification is modified by the m1A methyltransferases, or writers, such as TRMT6, TRMT61A, TRMT61B, TRMT10C, NML, and, removed by the demethylases, or erasers, including FTO and ALKBH1, ALKBH3. It is recognized by m1A-binding proteins YTHDF1, TYHDF2, TYHDF3, and TYHDC1, also known as “readers”. Additionally, we explore the intricate relationship between m1A modification and its regulators and their implications for the development and progression of specific types of cancer, we discuss how m1A modification can potentially facilitate the discovery of novel approaches for cancer diagnosis, treatment, and prognosis. Our summary of m1A methylated adenosine modification detection methods and regulatory mechanisms in various cancers provides useful insights for cancer diagnosis, treatment, and prognosis.

Video Abstract

**Supplementary Information:**

The online version contains supplementary material available at 10.1186/s12964-023-01401-z.

## Introduction

Classical genetics encompasses alterations in gene function arising from variations in gene sequence, such as gene mutations, thereby yielding inheritable modifications in phenotype. In contrast, epigenetics pertains to heritable changes in gene function that do not entail modifications in the DNA sequence of a gene, ultimately giving rise to phenotypic alterations [1]. Notably, modifications of DNA, RNA, and histones have been identified as influential factors in shaping phenotypes. While the discovery of RNA modifications dates to earlier times, they remain inadequately comprehende [2]. Historically, the field of epigenetics has predominantly focused on DNA and histone modifications. It is only in recent years that RNA modification has garnered significant attention and become a subject of extensive investigation [3–5].

RNA modification is a prevalent post-transcriptional regulatory mechanism present in various RNA types, such as messenger RNA (mRNA), transfer RNA (tRNA), ribosomal RNA (rRNA), and long non-coding RNA (lncRNA) [5–8]. RNA modifications encompass diverse chemical alterations, including methylation, hydroxymethylation, acetylation, and others. Among these, methylation modifications have been subject to the most extensive research, including N6-Methyladenosine (m6A) [8, 9], N1-methyladenosine (m1A) [10], and 5-methylcytidine (m5C) [11], each exerting distinct functions within different biological contexts. Historically, the primary focus was on m6A modifications [12], with limited attention directed towards other modification types, including m1A, particularly the roles of m1A.

m1A refers to a methylation modification occurring at the first adenosine position within RNA, carrying a positive charge under physiological conditions. Positioned at the Watson-Crick base-pairing interface, m1A exerts influence over base complementarity and RNA–protein interactions, thereby impacting transcription and translation processes [13]. m1A assumes a pivotal role in the pathogenesis of human diseases, with a particularly strong correlation to cancer development. Previous studies have shown that RNA methylation modification, specifically m1A, is closely associated with cancer-related processes such as proliferation, apoptosis, metabolism, and cell cycle regulation. For example, it contributes to tumorigenesis by perturbing the stability of relevant RNA molecules and further fuels cancer progression by modulating the translation of target mRNA, among other mechanisms. It also has implications for the diagnosis, prognosis, and treatment of cancer patients [14]. Recent research has increasingly demonstrated the involvement of “writer,” “reader,” and “eraser” regulators of m1A in regulating cancer occurrence and progression [3, 5, 10]. A few researchers have utilized these regulators for cancer detection, prognostication, clinical staging, and as targets for drug-based disease treatment. A comprehensive exploration of m1A relies heavily on the development of detection methodologies, which have evolved from traditional chemical analysis to contemporary high-throughput sequencing methods. This review provides a comprehensive overview of m1A detection methods and examines their respective strengths and weaknesses.

## m1A detection methods

As science and technology advance, methods for detecting methylation modifications are continually evolving. Initially, traditional detection approaches encompassed two-dimensional thin-layer chromatography (2D-TLC), high-performance liquid chromatography (HPLC), liquid chromatography-mass spectrometry (LC-MS), and primer extension. LC-MS and primer extension techniques frequently serve as complementary assays to validate novel experimental approaches. Subsequently, high-throughput sequencing methods have emerged, enabling precise identification of modification sites and stoichiometry on transcripts. Similar to the detection of m6A modifications, strategies exploiting the distinctive chemical properties of m1A to render it detectable after reverse transcription have been developed. These approaches involve altering the identity of the modified base or specifically attaching large residues to the modified base. Consequently, such modifications lead to premature truncation or misincorporation during reverse transcription, facilitating the inference of modification sites. High-throughput sequencing methodologies encompass various modalities for the detection of RNA modifications. One of the methods is α-ketoglutarate-dependent dioxygenase (AlkB)-facilitated RNA methylation sequencing (ARM-seq), which is based on ALKB enzyme-mediated demethylation. During the library preparation stage, this approach can elucidate modification sites that might otherwise elude detection due to potential interference by RNA containing modified nucleosides during reverse transcription. Other methods are antibody-based enrichment techniques, such as methylated RNA Immunoprecipitation sequencing (MeRIP-seq), (or m1A-seq) and m1A sequencing technique combining antibody enrichment and specific enzymatic reaction (m1A-ID-seq). Both of these methodologies incorporate additional strategies aimed at enhancing the reliability and resolution of the assay. The former method converts rt-interfering m1A to rt-silencing m6A through a chemically assisted reaction, while the latter employs RNA/DNA demethylase to eliminate the m1A modification after immunoprecipitation. Both methods infer the presence of m1A by analyzing peak values [15]. Furthermore, a single-base resolution technique called misincorporation-assisted profiling of m1A (m1A-MAP-seq), which is based on the thermostable group II intron reverse transcriptase (TGIRT) method, combines an antibody-mediated pre-enrichment step with an in vitro demethylation step. Substituting conventional reverse recruitment with TGIRT and superscript (SS) leads to m1A-seq-TGIRT with a higher false incorporation rate and m1A-seq-SS with a higher truncation rate. The more contemporary approaches, IP for immunoprecipitation sequencing with an anti-m1A antibody (m1A-IP-seq) and spiked in synthetic m1A oligonucleotides with various m1A fractions (m1A-quant-seq), offer improvements by replacing and enhancing the demethylation step [15, 16] (summarized in Table 1).

**Table 1:** Overview of m1A detection methods

Detection method	Feature	Advantage	Drawback	Function	Article source
2D-TLC	differential retention values(Rf values)	ease and cheap	hydrolysis and 32P labeling steps may have bias	–	[17]
HPLC	differential retention, enzymatic RNA digestion	faster no radiolabeling	loss of sequence information	analysis of modifications with high abundance	[17, 18]
LC-MS	HPLC, mass Spectrometry	high accuracy and sensitivity	loss of sequence information	detect and quantify the m1A level in mRNA	[15, 17, 19]
Primer-extension	block base pairing compared cDNA bands	Precise modification position	RNA targets of high abundance and existing sequence knowledge	validate the new detection technique	[17]
ARM-Seq	ALKB treatment	sensitive and accuracy	–	Assay escape modification, Analysis of RNA modification and processing sequences	[20]
m1A-ID-seq	RNA Immu-opreipitaion with ALKB assisted	no cross-reactivity	m1A was difficult to obtain, rely on specific m1A antibody	the first trans-criptomewide characterization of m1A	[21]
MeRIP-seq-or-m1A-seq	Dimroth rearrangement, immune-oprecipitation	low mutation rate, lower mismatch rate	rely on specific m1A antibody	–	[22]
m1A-MAP-seq	Immun-oprecipitation, AlkB treatment, TGIRT-mediated RT, ligation-based strand-specific library preparation protocol	Excellent readthrough efficiency and relatively high mutation frequency	TGIRT Under-estimated the m1A level, the sequence context of RNA affect the mutation rate	identify m1A modification at mRNA cap, and GUUCRA tRNA-like sequencemotif	[6]
m1A-IP-seq	ALKB treatment, immun-oprecipitation, Differential abundance analysis, RT-1306-mediated RT mutation	capture robustly mutation signature, good reproducibility, wide transcriptome coverage, high alignment rate to the genome	data re-producibility worse, false negatives, disable the detection of potential sites at or near the 5′-cap	discovered hundreds of new m1A sites in human mRNA	[6]
m1A-quant-seq	ALKB treatment spike-in RNA RT1306-mediated RT mutation	Mutation signatures and sensitivity to AlkB treatment are robustly observed	false negatives result, disable the detection of potential sites at or near the 5′-cap	estimate m1A stoichiometries at individual sites in the transcriptome	[6]
m1A-seq-TGIRT	Immun-oprecipitation, Dimroth rearrangement, TGIRT-mediated RT	identifying individual modified bases, higher misincorporation rates, not require adapter ligation	low truncation rates, low RT activity	redefinition of m1A genome-wide distribution	[6, 23, 24]
m1A-seq-SS	immunoprecipitation, Dimroth rearrangement, SS-mediated RT	identifying individual modified bases, premature truncations	–	–	[23]

### 2D-TLC

This technique is employed to detect the presence of m1A by separating nucleotides based on their differential retention values (Rf values) and subsequently comparing them with established nucleotide controls. The specific positioning of nucleotides depicted in this chromatographic approach is influenced by factors such as temperature, cellulose plate, solvent concentration, and the number of utilization cycles. Due to its user-friendly nature and cost-effectiveness, this method is the preferred choice of most researchers [17].

### HPLC and LC-MS

Uziel et al. compared the HPLC method to the prior hydrolysis of nucleic acids to nucleotides and hydrolyzed nucleic acids to nucleosides. High-resolution liquid chromatography, employing a small-diameter cation exchange resin and monitored by an ultraviolet spectrophotometer system, can be used to detect and analyze nucleotides under conditions of high line speed and pressure [18]. HPLC proves advantageous over 2D-TLC due to its speed and lack of necessity for radiolabeling. Additionally, it facilitates the separation of nucleotides based on their distinct Rf values and enzymatic hydrolysis for the analysis of RNA modifications. Nevertheless, HPLC is primarily suited for the examination of abundant RNA modifications. However, LC-MS, which offers greater sensitivity and the capability to detect individual nucleotides, initially hydrolyzes nucleic acids into nucleotides and subsequently utilizes LC/MS, or reverse-phase HPLC. It constitutes the coupling of liquid chromatography and mass spectrometry. However, LC-MS faces two limitations: firstly, it relies on the complete digestion of nucleosides; secondly, the detection and interpretation of low-abundance modifications prove challenging, with high levels of modifications often being overestimated [7, 15, 17, 19].

### Primer extension

Although all three of the aforementioned methods can be utilized for detecting m1A modifications, they entail enzymatic hydrolysis, resulting in the partial loss of RNA sequence integrity. With advancements in medicine, an increasing number of RNA modification sites are being linked to the onset and progression of diseases. Consequently, the demand for accurate m1A site detection has risen significantly. To address this, a more comprehensive method for discerning m1A modification—the primer extension method—has been developed. This technique obstructs complementary base pairing, truncating the synthesis of cDNA containing m1A modifications. Subsequently, it is compared with a known cDNA library to glean m1A modifications. Nevertheless, this approach is best suited for RNA modifications characterized by prominent peaks and necessitates prior knowledge of the corresponding DNA sequence [17].

### High-throughput sequencing approaches

#### ARM-seq

**Fig. 1:**
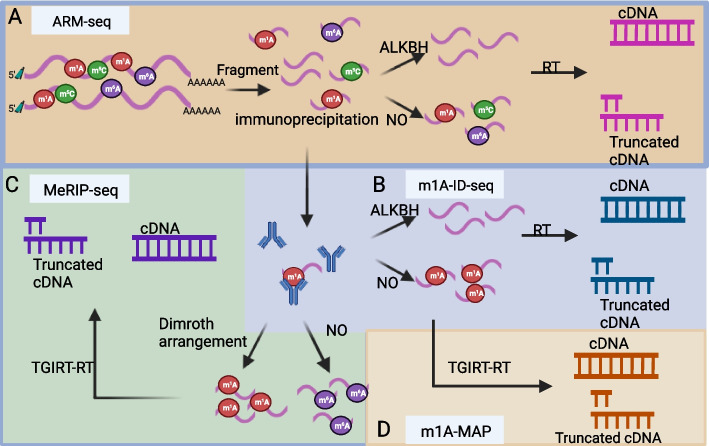
The techniques used for progressive renewal include ALKBH, immunoprecipitation, TGIRT-RT, and Dimroth arrangement. **A**. ARM-seq (ALKBH). **B**. m1A-ID-seq (ALKBH, immunoprecipitation). **C**. MeRIP-seq (ALKBH, immunoprecipitation, TGIRT-RT, and Dimroth arrangement). D. m1A-MAP (ALKBH, immunoprecipitation, and TGIRT-RT)

ARM-seq involves the treatment of RNA with ALKB demethylase from *Escherichia coli* prior to reverse transcription. Following reverse transcription, the precise location of m1A methylation within RNA is determined through differential abundance analysis (Fig. 1A). These findings indicate the presence of numerous m1A modifications in human tRNA. ARM-seq can enable the detection of methylated small RNAs originating from tRNAs. Additionally, it more than doubles the proportion of small RNA sequencing reads attributed to tRNA genes, from 6.9 to 15.1%. This enhancement facilitates a more comprehensive exploration of the association between tRNA and various disease states in humans. Furthermore, ARM-seq effectively identifies methylation-modified pre-tRNA and mitochondrial RNA and predicts the methylation status of adenosine at position 58 in mature tRNA. Consequently, ARM-seq holds promise as a valuable tool for disease detection [20].

### m1A-ID-seq

After RNA fragmentation, the extracted RNA is immunoprecipitated using an anti-m1A antibody. The resulting cDNAs are then divided into two portions: one portion is subjected to demethylation and reverse transcription to generate full-length cDNA, while the other is subjected to reverse transcription to produce truncated cDNA. Subsequently, differential abundance analysis is conducted on these two portions to ascertain the specific locations of m1A modifications (Fig. 1B). This approach employs an antibody with high specificity for m1A, thereby minimizing any potential cross-reactivity with m6A and facilitating more efficient purification of m1A-modified RNA. In addition, *E. coli*-derived ALKB is utilized to demethylate the RNA. By employing m1A-ID-seq technology, one can identify known m1A modification sites, including those within rRNA, as well as 901 novel m1A modification sites within the human transcriptome. Furthermore, the m1A peak is predominantly enriched in proximity to the 5′ untranslated region (5′-URT) and the start codon [21].

### MeRIP-seq

After RNA fragmentation, immunoprecipitation is performed using an anti-m1A antibody. Following elution, the RNA is divided into two fractions: one fraction is subjected to direct sequencing, while the other is subjected to rearrangement in an alkaline environment to convert m1A to m6A before sequencing. By comparing the sequencing results of these two fractions, the relative abundance of m1A can be determined (Fig. 1C). Utilizing the Dimroth rearrangement method, a lower mutation rate and mismatch rate during reverse transcription can be achieved, thereby enhancing the precision and comprehe m1A-map nsiveness of m1A abundance detection [22].

### m1A-map

RNA is subjected to immunoprecipitation following anti-m1A treatment and subsequently divided into two fractions: one fraction is subjected to in vitro demethylation using ALKB, while the other fraction is left untreated. The methylated RNA is then subjected to TGIRT-mediated reverse transcription to generate full-length cDNA, followed by library preparation through a linked strand-specific approach for subsequent comparison (Fig. 1D). However, the unmethylated RNA is reverse transcribed, exploiting m1A’s inherent ability to induce transcription truncation and misincorporation, resulting in the generation of multiple truncated cDNA fragments. By aligning these truncated cDNAs with the full-length cDNA library produced during transcription, the precise location of the m1A modification can be determined. This method facilitates the identification of m1A modification sites within mRNA cap structures and the GUUCRA base sequence in tRNA [6].

### m1A-IP-seq

**Fig. 2:**
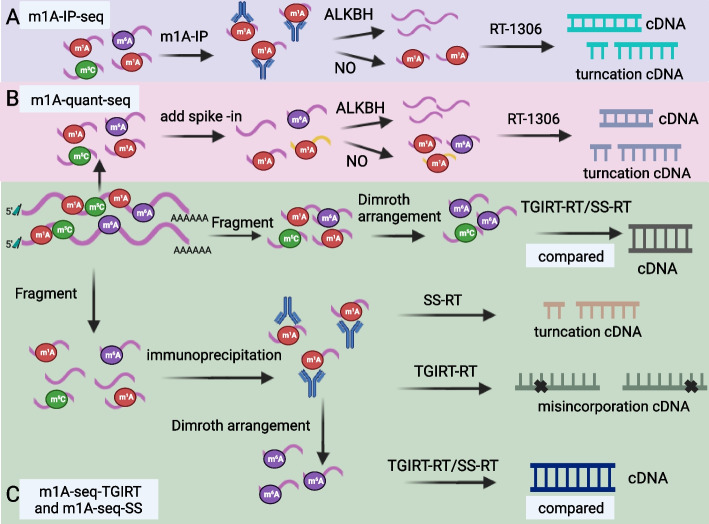
Detection of m1A modification. The stepwise updating techniques include more specific anti-m1A antibody IP, increasing the m1A fragment quant in RNA, and SS-RT, with a higher truncation rate, and TGIRT-RT, with a higher misincorporation rate, respectively. **A** m1A-IP-seq (IP). **B** m1A-quant-seq (quant). **C** m1A-seq-TGIRT and m1A-seq-SS (SS-RT and TGIRT-SS)

In this technique, the novel technology RT-1306 yields a 10-fold increase in full-length cDNA production and a higher ratio of reads to truncated products compared to TGIRT. Consequently, it enables the acquisition of more comprehensive cDNA. The sequencing method employed is similar to that of ARM-seq. Initially, RNA is subjected to treatment with the demethylase α-ketoglutaric acid-dependent dioxygenase (ALKB) from *E. coli*, followed by reverse transcription using RT-1306 to generate comparable cDNA. Subsequently, the specific position of m1A is ascertained through differential abundance analysis (Fig. 2A). However, due to the necessity for immunoprecipitation and demethylation processing, data reproducibility may be compromised. In addition, reliance on demethylation treatment may lead to false negatives, particularly when RNA methylation abundance is low or when the methylation site is situated within intricate structures that are impervious to enzymatic demethylation treatment. In the future, the development of a combination of enzymatic and chemical demethylation techniques will enhance sensitivity to these sites. The current data processing procedure mandates sequence alignment using soft clipping to circumvent potential errors arising from non-templated addition by reverse transcriptase (RT) However, this approach may render the detection of potential sites located at or in proximity to the 5′-cap ineffective [23].

### m1A-quant-seq

Small RNA is isolated from total RNA and subjected to DNase-mediated degradation and subsequent enrichment through polyA selection. Then, 100-nt fragments of RNA are screened. Subsequently, the fragments associated with m1A are directly incorporated into the RNA, thereby partitioning it into two fractions. One fraction is subjected to demethylation via ALKB treatment, followed by reverse transcription employing RT-1306 to generate cDNA encompassing m1A modifications. The other fraction, not subjected to any treatment, is directly reverse transcribed using RT-1306, yielding multiple truncated cDNAs. Differential abundance analysis allows for the precise identification of the specific m1A positions (Fig. 2B). The immunoprecipitation (IP) and quantification methods are employed to produce high-quality libraries of 250-bp fragments, characterized by a high alignment rate and broad transcriptome coverage. These methods demonstrate suitability for cell culture applications, with robust reproducibility of expressed transcripts across replicate experiments [23].

### m1A-seq-TGIRT and m1A-seq-SS

After DNAse digestion, RNA fragments of approximately 100 nucleotides in length are selected following polyA screening. Subsequently, the RNA is divided into two fractions: one fraction is subjected to immunoprecipitation using an anti-m1A antibody and is ligated at the 3′ end, followed by reverse transcription mediated by TGIRT. The other fraction is subjected to immunoprecipitation with an anti-m1A antibody and subjected to an alkaline environment to induce Dimroth rearrangement, converting m1A to m6A. Afterward, it too is ligated at the 3′ end and subjected to reverse transcription, either utilizing TGIRT or SS. The precise localization of m1A modifications can then be determined through differential abundance analysis (Fig. 2C). Research has revealed that, compared to the use of m1A-seq, the utilization of TGIRT results in a higher incorporation rate and lower truncation rate, whereas the use of SS yields a higher truncation rate but a lower false incorporation rate. Both methods exhibit enhanced sensitivity and specificity in detecting m1A modifications, leading to a reduction in false-positive rates and a more accurate estimation of m1A stoichiometry compared to previous approaches [23–25].

## Regulators of m1A modification

**Fig. 3:**
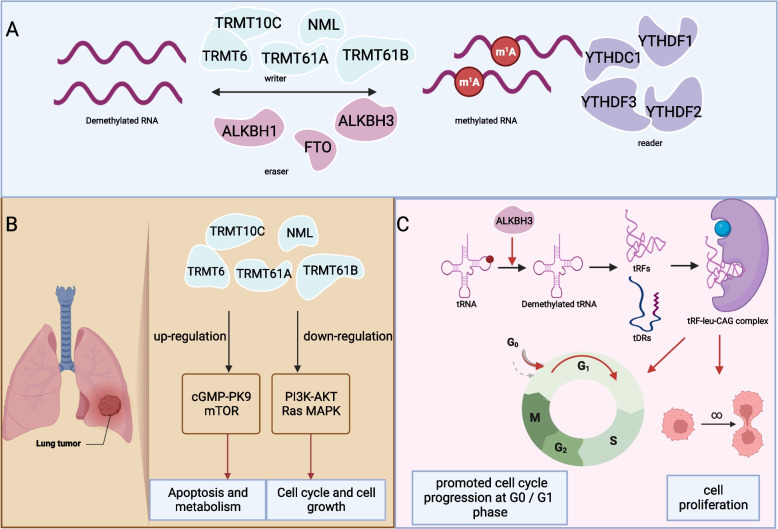
Regulators of m1A modification include writers, readers, and erasers. The expression of writers affects the occurrence of cancer. ALKBH3 regulates the proliferation of cancer through tDRs and tRFs. **A** Regulatory factors of RNA, including writers, erasers, and readers. **B** The regulatory mechanism of m1A in lung cancer (1) (writer). **C** The regulatory mechanism of m1A in lung cancer (2) (ALKBH3)

Similar to m6A modification, m1A modification has its own set of regulators, which encompass “writers,” “readers,” and “erasers.” The group responsible for m1A methylation, the “writers,” consists of tRNA methyltransferase 6(TRMT6), TRMT61A, TRMT61B, TRMT10C, and a nucleolar factor, nucleomethylin (NML) [24, 26–29]. The “readers” that recognize this methylation include (YTH) domain-containing proteins (YTHDF1), TYHDF2, TYHDF3, and TYHDC1. AlkB homolog 1 (ALKBH1), ALKBH3, and fat mass and obesity-associated protein (FTO) serve as the “erasers” responsible for demethylation (Fig. 3A). Each of these three regulatory classes will be elaborated upon below. The members of the ALKBH family, comprising nine members, including ALKBH1 to ALKBH8 and FTO, have evolved from prokaryotic DNA repair enzymes [30, 31]. Demethylation in this family relies on Fe^2+^ and α-ketoglutarate to initiate the dealkylation reaction, whereby a bound water molecule is replaced, allowing oxygen to bond with Fe [32]. Wang et al. reported direct binding of YTH domain proteins, namely YTHDF1, YTHDF2, and YTHDC1, to m1A-modified RNA oligonucleotides [6]. However, differing findings were reported by Kyung W. Seo and Ralph E. Kleiner et al., who corroborated and expanded on the binding of YTHDF1/2 to m1A. Nevertheless, the specific binding of YTHDC1 to m1A could not be observed in their studies [33].

### Writers

Through high-throughput sequencing, the presence of m1A modification has been identified in mRNA, tRNA, and rRNA. While mRNA displays a relatively low occurrence of m1A modification, tRNA exhibits a stable m1A modification in the neck loop region, characterized by a robust hairpin structure consisting of a 5-base pair (bp) stem and a 7-bp loop. The m1A modification in tRNA is attributed to the TRMT6/TRMT61A complex, and it is contingent upon the cloverleaf structure inherent to tRNA. Such mRNA modification requires a structure similar to the tRNA neck loop structure along with the GUUCRA sequence. Knockdown of the TRMT6/TRMT61A complex results in diminished m1A modification in the neck loop structure, whereas the overexpression of both subunits leads to an augmentation of m1A modification at corresponding positions. Notably, this effect is most pronounced at position 384 in cytoplasmic mRNAs, lncRNAs, and select tRNA sites. In human cells, the use of small interfering RNAs to knock down TRMT61A and TRMT6 has been observed to retard cell growth, suggesting that TRMT61A and TRMT6 play pivotal roles in influencing cell proliferation. TRMT61B encodes the mitochondria-specific tRNA methyltransferase responsible for catalyzing the m1A58 modification within RNA-Leu (UUR), tRNA-Lys, and tRNA-Ser (UCN) [27]. In addition, TRMT10C has been identified as the catalyst for the m1A modification at position 9 in mitochondrial ND5 mRNA. Knockdown of TRMT10C results in the loss of methylation at ND5:1374, whereas overexpression of TRMT10C increases methylation levels by 50%. These methylation levels exhibit tissue-specific variation and require precise regulation. Furthermore, it has been demonstrated that m1A modifications found within the Sequence coding for amino acids in protein (CDS) and 5′-UTR can impede the translation process. This inhibition may depend on ribosome scanning and translation mechanisms.

NML serves as a catalytic factor in the modification of 28S rRNA. The NML-dependent C-terminal region encompasses a Rossman-fold methyltransferase-like domain, which plays a pivotal role in the methylation of A1309 and A1136 on 28S rRNA in both humans and murine organisms. Depletion of NML leads to a reduction in the formation of the 60S ribosomal subunit without impacting protein synthesis. Additionally, it triggers the activation of the p53 pathway through its interaction with the RPL11/MDM2 complex. NML does not exert an influence on ribosomal deoxyribonucleic acid (rDNA) transcription under normal sugar conditions, nor does it affect the processing of precursor rRNA into its mature form [34].

### Erasers

Demethylation of target tRNAs catalyzed by ALKBH1 leads to attenuated translation initiation and reduced utilization in protein synthesis. ALKBH1 predominantly targets the m1A modification on tRNA, possessing a tRNA-binding domain employed by tRNA ligases for tRNA recognition. ALKBH1 primarily functions by recognizing the neck loop structure within tRNA. The m1A modification at position 58 in tRNA serves as the primary recognition site for ALKBH1. ALKBH1 modulates protein synthesis through the modification of 58m1A on specific RNA molecules. Notably, ALKBH1 selectively modifies m1A and does not interact with N7-methylguanosine(m7G), m5C, or 3-methylcytidine (m3C) modifications. Additionally, ALKBH1 exhibits limited recognition of m1A in mRNA. m1A-hypermodified tRNA-Val (mAC), tRNA-His (GUG), and tRNA-Gly (GCC) preferentially bind to translationally active polysomes. Overexpression of ALKBH1 results in reduced methylation levels of tRNA-His (GUG) and tRNA-Gly (GCC), accompanied by decreased tRNA quantities. ALKBH1 knockout induces an upsurge in tRNA-iMet, thereby promoting translation initiation and the overall translation process. Methylated tRNA molecules are more likely to be recruited by ribosomes for translation [35].

A study conducted by Zhuo Jia Chen et al. yielded valuable insights into ALKBH3, highlighting its possession of a tRNA-binding domain similar to ALKBH1, suggesting a potential tRNA recognition function. However, ALKBH3 exhibits unique specificity for tRNA recognition, effectively engaging with most tRNAs, with the notable exceptions of tRNA-iMet and tRNA-Phe (GAA). ALKBH3 effectively modulates the stability of 58m1A. Moreover, their investigation revealed the ubiquitous expression of ALKBH3 in cancer cells, where it can act as a catalyst for cancer progression, generating tRNA fragment (tDRs) that facilitate ribosome assembly while inhibiting apoptosis in cancer cells [36]. ALKBH3 exhibits a pronounced preference for short sequences and single-stranded DNA/RNA, a characteristic corroborated by other studies [7, 22, 35, 37].

FTO, similar to ALKBH1, functions as an m1A demethylase with a greater preference for the neck loop structure within tRNA over unstructured RNA. It also affects translation through tRNA demethylation, consistent with the pathway through which ALKBH1 exerts its translational influence [38].

### Readers

TYHDF1, TYHDF2, TYHDF3, and TYHDC1 are all members of the YTH family and can directly bind to m1A RNA. In YTHDF2, Trp432, a conserved residue within the hydrophobic pocket of the YTH domain, is crucial for m6A recognition, and it is a prerequisite for N-6 binding as well as m1A recognition [39].

YTHDF3 directly binds to m1A, exerting a negative regulatory effect on trophoblast cell invasion and migration. This is achieved through the promotion of mRNA degradation, which subsequently leads to reduced production of the insulin-like growth factor receptor 1 protein. Furthermore, YTHDF3 inhibits the downstream signaling pathway of matrix metallopeptidase 9, ultimately diminishing the migration and invasion capabilities of trophoblast cells [40].

Using YTHDF1 as the target transcript for analysis instead of considering all methylated mRNAs, researchers have revealed a role of YTHDF1 in enhancing translation in HeLa cells. YTHDF1 and YTHDF2 interact with distinct protein partners and assemble into dissimilar higher-order structures. Concurrent knockdown of all three YTHDF proteins leads to an augmentation in cellular P-body formation and a global stabilization of mRNA molecules, irrespective of their methylation status [41].

## m1A and cancer

The exploration of m1A modification in cancer research has consistently demonstrated its impact on various cellular processes, including cell proliferation, metabolism, and apoptosis. Therefore, it has found extensive utility in the realms of disease diagnosis, treatment, and prognosis. Notably, in the context of cancer, numerous studies have elucidated the relationship between m1A regulators and a diverse spectrum of malignancies. These regulators play pivotal roles in the initiation, therapeutic strategies, and prognostication of diseases such as liver cancer, prostate cancer, glioma, colorectal cancer (CRC), bladder cancer (BC), lung cancer, and pancreatic cancer (PC) (summarized in Table 2).

**Table 2:** Overview of m1A and its role in cancer mechanisms

Cancer	Regulator	Pathway	Function	Resource
LUAD	Writer ALKBH3	cGMP PKG mTOR Signaling, tRF-leu-CAG	cell apoptosis and tumor metabolism, cell cycle progression and proliferation	[47–50]
Breast cancer	ALKBH3 TRMT6	CSF-1 miR-191-5p	Invasion, proliferation and migration	[57, 58]
Colorectal-cancer	ALKBH1 Writer	SMAD7, MFAP2-CLK3	metastasis and prognosis, development, AJCC stage	[68–70]
Pancreatic-cancer	ALKBH1, ALKBH3, TRMT61B, Writer YTHDC1, YTHDF2	mTOR and ErbB, EMT, TGF –β and mTOR C1 signaling	poor survival, proliferation, invasion and migration	[75–77]
Hepatocellular-carcinoma	TRMT6/TRMT61A, TRMT10C, YTHDF1	Hedgehog PPAR Signaling, PI3K/Akt signaling pathway	CSCs self-renewal and tumorigenesis, process of proliferation and apoptosis resistance	[68, 80–82]
Glioma-	ABCC3, ALKBH1, ALKBH3, TRMT61B, TRMT6, TRMT10C, TRMT61A	–	Proliferation and resistance	[88–92]
Bladder-cancer-	TRMT6/TRMT61A, ALKBH3	tRF-3- UPR, NOX-ROS signaling	Tumorigenesis, tumor angiogenesis and invasiveness	[97, 98]

### Lung adenocarcinoma (LUAD)

Lung cancer stands as one of the most prevalent and deadliest malignancies globally [42, 43]. This disease is categorized into two primary subtypes: non-small cell lung cancer (NSCLC) and small cell lung cancer. NSCLC can be further classified into LUAD, squamous cell carcinoma, and large cell carcinoma.

NSCLC, constituting approximately 80% of all lung cancer occurrences, has attracted research interest extending beyond the exploration of EGFR, KRAS, and ALK genes. Particularly in recent years, epigenetic RNA modifications have garnered attention in the context of NSCLC [44–47]. Some studies have divided patients with LUAD into two distinct cohorts: the high writer-score group and the low writer-score group. In the high writer-score group, heightened activation of the cGMP/PKG and mTOR signaling pathways has been observed, with notable associations to apoptosis and tumor metabolism. In contrast, the low-score group has exhibited significant activation of the PI3K/AKT, Ras, and MAPK signaling pathways, aligning with cell cycle regulation and normal cell growth (Fig. 3B). These findings suggest a potential relationship between RNA modification writer-score, tumor prognosis, and the tumor microenvironment. In addition, writer-score demonstrates promise in predicting the prognosis of patients with NSCLC undergoing neoadjuvant anti-PD-1 therapy [48]. ALKBH3, a demethylase targeting m1A, has also emerged as a participant in carcinogenesis. Its heightened expression in LUAD has been correlated with post-recurrence survival [49]. ALKBH3 plays a role in the demethylation of tRNA, resulting in an increased abundance of small RNA tDRs and tRFs derived from tRNA. This, in turn, elevates the translation rate while inhibiting cell apoptosis [36]. The tRF/Leu/CAG complex has been shown to promote cell cycle progression at the G0/G1 phase, inducing cell proliferation in NSCLC [50, 51] (Fig. 3C).

### BC

BC ranks as the tenth most prevalent form of cancer globally, with 573,278 cases in 2020. Notably, the incidence of BC in males is approximately four times higher than in females. Despite the significant advancements in medical technology, there has been limited progress in reducing the incidence and mortality rates associated with this disease [52]. In 2022, BC emerged as the second most common malignant tumor within the urinary system, accounting for 17,100 out of the 81,180 new cases reported. Despite the availability of a wide array of treatment modalities, the prognosis for BC remains unsatisfactory [53–56].

**Fig. 4:**
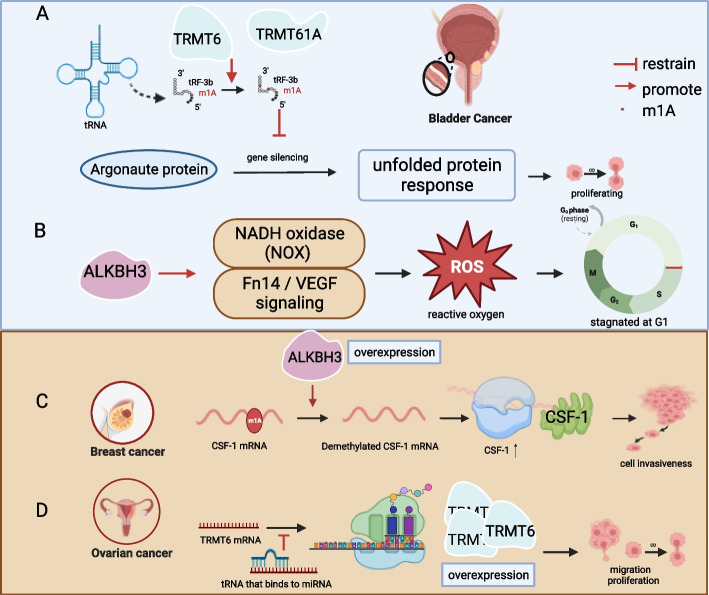
TRMT6/61A regulates BC and affects UPR, and ALKBH3 regulates the NOX/ROS signaling axis to affect tumor angiogenesis and invasiveness. **A** TRMT6/61A and BC (UPR). **B** ALKBH3 and BC (NOX/ROS). The m1A regulator ALKBH3 regulates the development of breast cancer and ovarian cancer through CSF-1. TRMT6 regulates the development of ovarian cancer through miR-191-5p. **C** The regulatory mechanism of m1A in ovarian cancer and breast cancer (ALKBH3). **D** The regulatory mechanism of m1A in ovarian cancer (TRMT6)

In recent years, the advancement of our understanding of BC has been significantly augmented by increased research into RNA modification. It has come to light that elevated levels of m1A modification in BC coincide with dysregulation of genes associated with the unfolded protein response (UPR). Specifically, in bladder urothelial carcinoma, the heightened m1A modification of tRF-3b is catalyzed by the TRMT6/61A complex, resulting in the dysregulation of tRF-targeted mRNA. This dysregulation subsequently impacts cancer progression by influencing the UPR (Fig. 4A). Therefore, UPR holds promise as a potential therapeutic target in the future [57].

Beyond the writers of RNA modification, ALKBH3, functioning as an eraser of m1A, also plays a role in promoting the development of human urothelial carcinoma. It does so by modulating NADH oxidase (NOX) and Fn14/VEGF signaling pathways. This effect is contingent on the generation of reactive oxygen species (ROS), which can induce cell cycle arrest at the G1 phase [58]. Notably, while NOX/ROS signaling does not exert a significant influence on tumor angiogenesis and invasiveness in human BC, it does promote angiogenesis in ovarian cancer [59, 60] (Fig. 4B). Zhang Yang et al. validated that mutations and copy number alterations in the m1A writer genes may be associated with BC. Furthermore, they identified TRMT61A as a poor prognostic factor for BC. Notably, the poor-prognosis writer-score-high group exhibited enrichment of oncogenic, proliferative, and apoptotic pathways [61]. Alterations in the CNV of writers also contribute to the distinctions between patients with BC and healthy individuals. Furthermore, the RNA modification “writers” score(RMS) has been positively correlated with the malignancy of BC [62].

### Ovarian cancer and breast cancer

In 2020, breast cancer emerged as the most prevalent malignancy globally, with 2.3 million new cases, surpassing lung cancer. It constitutes 24.5% of all cancer cases in females. Notably, Asia accounted for nearly half of breast cancer diagnoses (45.4%) [63]. The contemporary therapeutic repertoire for breast cancer includes surgery, chemotherapy, radiotherapy, hormone therapy, and targeted interventions [64]. However, the efficacy of these modalities remains limited, necessitating a pressing exploration of novel and more radical treatment strategies [65]. Ovarian cancer, among gynecologic cancers, assumes the distinction of being the deadliest. In 2016, China recorded an estimated 57,200 new ovarian cancer cases, resulting in 27,200 fatalities. Due to the absence of reliable early detection methods, the majority of diagnoses occur at advanced stages, marked by poor prognoses and elevated recurrence rates. While significant strides have been taken in ovarian cancer treatment, its overall survival rate remains low [66]*.* The incidence and mortality rates of ovarian cancer in China have exhibited an upward trajectory over the past three decades, with this trend anticipated to persist over the next 30 years [67].

The modulation of breast cancer through RNA m6A modification has been the subject of extensive research. This modification orchestrates the proliferation, invasion, and metastasis of breast cancer by effecting changes in diverse signaling pathways [68]. However, existing research on the influence of m1A in breast cancer remains insufficient. In recent years, studies on RNA modifications in both ovarian and breast cancer have revealed that Macrophage Colony Stimulating Factor 1(CSF-1) is associated with unfavorable prognoses in both cases. Furthermore, the expression of CSF-1 is subject to post-transcriptional modification, particularly RNA modification. Specifically, the demethylation of RNA by ALKBH3 regulates the degradation of CSF-1 mRNA and controls its half-life. In summary, the overexpression of ALKBH3 in ovarian and breast cancer leads to a reduction in m1A levels within CSF-1 mRNA. This reduction prolongs the mRNA’s half-life, enhancing its stability and consequently promoting cancer cell invasiveness. This alteration does not affect cancer cell proliferation or migration capabilities [69] (Fig. 4C). Overexpression of TRMT6 augments breast cancer cell proliferation and migration. Jiang Zhao et al. demonstrated that high TRMT6 expression in patients with ovarian cancer correlates with poor prognoses compared to those with low TRMT6 expression. The adverse effects of high TRMT6 expression can be mitigated through targeted regulation of TRMT6 by upstream miR-191-5p [70]. MicroRNAs (miRNAs) serve as regulatory factors that inhibit translation and protein expression by binding to mRNA. They play pivotal roles in the regulation of cell proliferation and migration (Fig. 4D). Among miRNAs, miR-191-5p exerts a regulatory function in various other cancers, demonstrating distinct roles in different contexts. For instance, it has been implicated in the suppression of miRNA expression in gastric cancer, renal epithelial cancer, and other malignancies, while promoting osteosarcoma development through the targeting of Recombinant Early Growth Response Protein 1(EGR1) [70–73].

### CRC

CRC ranks third in incidence and second in mortality among all cancers [74, 75]. Some patients are diagnosed with distant metastasis at the time of diagnosis [53]. In recent years, significant progress has been achieved in CRC treatment; however, the 5-year survival rate remains notably low [76]. The investigation of RNA modification has revealed that RNA regulators can influence the onset and progression of CRC [77, 78].

**Fig. 5:**
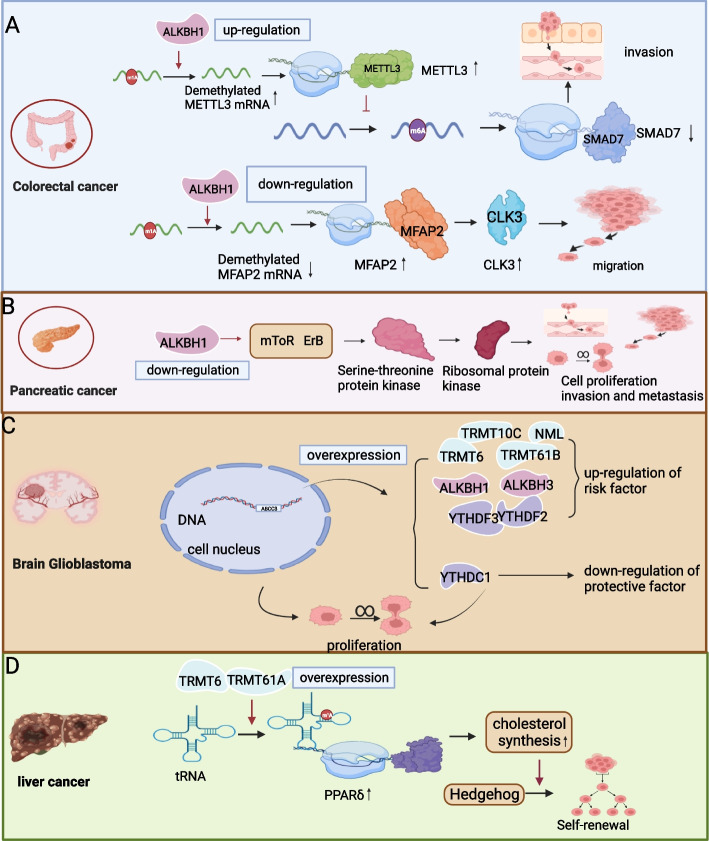
ALKBH1 promotes CRC development by regulating SMAD7 and MFAP2, whereas ALKBH1 plays a robust regulatory role in PC. **A** The regulatory mechanism of m1A in CRC. **B** The regulatory mechanism of m1A in PC. glioma progresses through the upstream *ABCC3* gene, whereas HCC progresses through a downstream signaling pathway regulated by TRMT61/TRMT6T. **C** The ABCC3 gene controls the proliferation of cancer cells by regulating the m1A regulator. **D** The regulatory mechanism of m1A in HCC

ALKBH1 is notably overexpressed in CRC tissues and is associated with metastasis and prognosis in patients with CRC. These findings align with those reported by Yue Shui Zhao et al., who observed that m1A regulators, except TRMT61A, which is typically expressed in esophageal cancer and colon adenocarcinoma, are overexpressed in all gastrointestinal tumors. Furthermore, their study established a negative correlation between ALKBH1 overexpression and overall survival in CRC. In contrast, a diminished expression of ALKBH3 in CRC correlates with poorer overall survival [79]. Overexpression of ALKBH1 leads to increased expression of methyltransferase 3 (METTL3). Consequently, this upregulation of METTL3 results in enhanced methylation of drosophila mothers against decapentaplegic protein 7(SMAD7), ultimately leading to its downregulation. Consequently, these molecular events promote the migration and invasion of CRC cells, contributing to CRC development and predicting an unfavorable prognosis (Fig. 5A). ALKBH1 exhibits distinct roles in various diseases. In PC, low ALKBH1 expression is linked to a dismal prognosis, while in most other tumors, ALKBH1 typically functions as an oncogene. Examples include glioma and gastric cancer [80].

Microfibrillar-associated protein 2(MFAP2), which is closely associated with lymph node metastasis, distant metastasis, and the advanced AJCC stage in CRC, exhibits high expression levels in patients with CRC, influenced by m1A regulation (Fig. 5A). Specifically, m1A upregulates MFAP2 expression in CRC cells. Knocking down ALKBH1 leads to increased m1A methylation levels and elevated MFAP2 mRNA expression. MFAP2 operates through its downstream target, CDC-like splicing factor kinase 3 (CLK3), a member of the CLK family possessing bispecific kinase activity [81]. MFAP2, an extracellular matrix glycoprotein, plays a pivotal role in microfibril assembly, elastin production, and tissue homeostasis [82]. In addition, MFAP2 promotes the migration and invasion of gastric cancer cells by enhancing the PI3K/AKT pathway [83]. CLK3 also fosters the migration and invasion of CRC cells, with studies indicating its involvement in cholangiocarcinoma and liver cancer invasion [84, 85].

### PC

PC ranks as a prevalent malignancy in the digestive system and currently stands as the seventh leading cause of cancer-related mortality, as reported by the International Agency for Research on Cancer. Despite ongoing advancements in chemotherapy and immunotherapy, their effectiveness remains suboptimal. Surgical intervention remains the primary mode of treatment, underscoring the imperative for the discovery and development of more efficacious therapeutic strategies. Within the realm of RNA modification research, the application of m1A modification has emerged as a promising avenue for PC treatment.

Shen P, Yang T, and Chen Q et al. reported a significant upregulation of ALKBH3 in patients with PC [86]. The diminished expression of ALKBH1 is associated with an unfavorable prognosis in PC. Through Kyoto Encyclopedia of Genes and Genomes (KEGG) pathway analysis, Qingyuan Zheng and Xiao Yu et al. incorporated m1A regulatory genes, revealing that these regulatory genes were predominantly implicated in 24 signaling pathways, notably the mTOR and ErbB pathways. These findings underscored a substantial correlation between ALKBH1 and both mTOR and ErbB, with all regulated genes except for TRMT61A. The mTOR pathway, downstream of PI3K/AKT, directs serine-threonine protein kinase, thereby modulating tumor cell proliferation, invasion, and metastasis via ribosomal protein kinase activation (Fig. 5B). Missense mutations and Copy Number Variation (CNVs) in m1A regulators also hold significance in PC. Zheng Qing Yuan et al. identified YTHDC1 as the m1A reader with the highest mutation frequency and YTHDF2 as the m1A reader with the highest CNV in patients with PC. This suggests that alterations in these regulatory genes may potentially impact the ATM signaling pathway, with CNVs of m1A linked to the prognostic outcomes of patients with PC [87].

ALKBH3, a demethylase, exhibits elevated expression levels in patients with PC [86]. An investigation of the relationship between mutations in RNA writer genes and PC showed that the high writer modification (WM) -score subgroup displayed an unfavorable prognosis, along with activation of oncogenic pathways such as EMT, TGF-β, and mTORC1 signaling pathways. Conversely, the low writer-score subgroup exhibited a more favorable prognosis. In patients with PC, the CNVs of TRMT61B showed an increase, resulting in higher TRMT61B expression compared to writers with unaltered CNVs [88]. In an examination of lncRNA methylation in PC, Hu Yu Quan et al. noted a significant elevation in resting memory CD4 T cells, M0 macrophages, and activated dendritic cells in the high-risk group compared to the low-risk group. Furthermore, it is suggested that modifications of these lncRNAs can serve as potential markers for early PC diagnosis, prognosis assessment, and identification of potential biological targets for immune response modulation [89].

### Glioma

Gliomas, including low-grade gliomas and glioblastomas (GBMs), represent the most prevalent and lethal malignancies in the central nervous system. Although surgery combined with radiotherapy and chemotherapy remains the primary treatment approach, the prognosis remains suboptimal. Despite undergoing clinical phase 3 trials, immune checkpoint inhibitor therapy has failed to enhance patient survival rates. The tumor microenvironment and tumor heterogeneity are pivotal factors in this context [90–93].

Regulatory factors of m1A modification offer insights into prognosis and may be potential therapeutic targets. Specifically, NML, TRMT61B, TRMT6, TRMT10C, ALKBH1, ALKBH3, YTHDF2, and YTHDF3 exhibit upregulation, while YTHDC1 displays downregulation. These m1A modification regulators exert significant influence on glioma prognosis. Among them, NML, TRMT6, TRMT10C, TRMT61B, ALKBH1, ALKBH3, YTHDF1, YTHDF2, and YTHDF3 are considered risk factors, while YTHDC1 is considered a protective factor. These regulators can, in turn, be upregulated through the activation of the *ABCC3* gene. ABCC3, in turn, can alter the tumor microenvironment to induce drug resistance and promote tumor proliferation [94, 95] (Fig. 5C).

TRMT61A is also highly expressed in highly aggressive gliomas. Under hypoxic conditions, c-MYC is suppressed in GBM, resulting in the downregulation of TRMT61A [96]. In addition, ALKBH1 has been found to inhibit cell proliferation and extend the lifespan of mice with GBM; however, this effect has not been achieved through stabilization. Zhao Kai et al. categorized gliomas into cluster 1 and cluster 2 based on RNA epigenetic modifications. Their study revealed that the overall survival rate was significantly lower in cluster 1 than in cluster 2. Cluster 1 was enriched in cell cycle-related processes, primary immunodeficiency, cytokine-receptor interactions, extracellular matrix receptor interactions, and various glioma carcinogenic pathways [97]. Additionally, low expression of TRMT6 was observed in cancer tissues [98].

### Hepatocellular carcinoma (HCC)

HCC constitutes the predominant form of liver cancer, accounting for approximately 90% of liver cancer cases. Despite the utilization of targeted drugs in HCC treatment, the challenges posed by its heterogeneity and elevated recurrence rates continue to impede progress in precision medicine and prognosis. Several factors contribute to the heightened mortality rate associated with liver cancer, including atypical early manifestations that hinder early diagnosis and the liver’s rich blood supply, which facilitates metastasis [99]. We summarize certain RNA modifications associated with HCC and explore their precise impacts on the initiation and progression of this malignancy.

Notably, a significant elevation in m1A levels has been observed in the tissue tRNA of patients with HCC. The catalysis of m1A is facilitated by TRMT6/TRMT61A and is highly upregulated in patients with advanced HCC. Furthermore, Kaplan–Meier analysis conducted by Yue Shui Zhao et al. demonstrated a correlation between high expression of m1A-regulated genes and a poor prognosis in HCC [79].

The TRMT6/TRMT61 complex enhances peroxisome proliferator-activated receptor (PPARδ) translation by elevating methylation levels, thereby instigating cholesterol synthesis and activating the Hedgehog pathway. This ultimately results in the self-renewal of tumor stem cell (CSCs) and tumorigenesis. Cholesterol synthesis is essential for Hedgehog pathway activation, implying that targeting the TRMT6/TRMT61A complex could offer a therapeutic approach for HCC (Fig. 5D). Jianping Zhang, Jie Gao, et al. proposed that RNA methylation modification regulators could serve as potent biomarkers or potential therapeutic targets for HCC [100, 101]. TP53 is a well-established tumor suppressor, and its mutations are known to promote cancer development. These mutations are associated with m1A regulators, including TRMT6, TRMT61A, TRMT10C, and YTHDF1, all of which also function as predictors of cancer risk. Furthermore, these regulators exert their effects through the PI3K/AKT signaling pathway [102]. HCC research has highlighted that modulation of the PI3K/AKT signaling pathway can effectively regulate the processes of proliferation and resistance to apoptosis in HCC [102, 103].

## Conclusion

In this review, we elucidated the regulatory factors governing m1A, its detection methodologies, and its modification status in the context of cancer. This review primarily focused on the identification techniques for diverse m1A modifications and their implications for cancer. Specifically, we explored how alterations in the m1A modification status across various RNA molecules can influence the pathogenesis and progression of several cancer types, including liver cancer, BC, ovarian cancer, breast cancer, lung cancer, glioma, PC, and CRC. Furthermore, we explored the potential for establishing prognostic models based on the regulatory factors of m1A. These models can serve to grade the severity of patients’ diseases, predict patient outcomes, and even be employed as prospective therapeutic targets. Despite the availability of reasonably accurate m1A detection methodologies for monitoring its role in tumor development, the precise m1A modification sites remain elusive, particularly in cases such as CRC where m1A has been shown to regulate the MFAP2/CLK3 axis [82]. Therefore, it is imperative to undertake further research and development efforts to refine the accuracy of m1A detection techniques, enabling their versatile application. Additionally, fostering advancements in m1A detection methodologies may not only benefit cancer research but also facilitate the exploration of specific molecular targets where m1A exerts its influence in cancer treatment. This, in turn, can pave the way for the development of more effective cancer-targeting drugs, ultimately contributing to the extension of human life.

Despite the growing interest in m1A modification of RNA, further research should prioritize investigating the interplay between m1A and other modification types, such as m6A. It is crucial to explore the potential cross-linking effects between these modifications while studying m1A. Existing evidence has demonstrated that m1A modification can enhance the impact of m6A on RNA by leveraging its regulatory factor [104]. Consequently, uncovering the cross-linking effect with other modification types becomes imperative. Moreover, it is worth noting that ALKBH family proteins and FTO family proteins are co-regulatory proteins of both m1A and m6A modifications. Therefore, a comprehensive understanding of the combined influence of these shared regulatory factors on the two modification types within the same pathway necessitates further investigation.

## Data Availability

Not applicable.

## Abbreviations

m1A: N1-methyladenosine
mRNA: messenger RNA
tRNA: transfer RNA
rRNA: ribosomal RNA
lncRNA: long non-coding RNA
m6A: N6-Methyladenosine
m5C: 5-methylcytidine
2D-TLC: two-dimensional thin-layer chromatography
HPLC: high-performance liquid chromatography
LC-MS: liquid chromatography-mass spectrometry
ARM-seq: α-ketoglutarate-dependent dioxygenase (AlkB)-facilitated RNA methylation sequencing
MeRIP-seq: methylated RNA Immunoprecipitation sequencing
m1A-ID-seq: m1A sequencing technique combining antibody enrichment and specific enzymatic reaction
m1A-MAP-seq: misincorporation-assisted profiling of m1A
TGIRT: thermostable group II intron reverse transcriptase
SS: superscript
Rf values: differential retention values
ALKB: α-ketoglutaric acid-dependent dioxygenase
RT: reverse transcriptase
IP: immunoprecipitation
TRMT6: tRNA methyltransferase 6
NML: nucleomethylin
YTHDF1: YTH domain-containing proteins
ALKBH1: AlkB homolog 1
FTO: fat mass and obesity-associated protein
CDS: Sequence coding for amino acids in protein
rDNA: ribosomal deoxyribonucleic acid
tDRs: tRNA fragment
tRFs: tRNA fragment
CRC: colorectal cancer
BC: bladder cancer
PC: pancreatic cancer
LUAD: Lung adenocarcinoma
ABCC3: ATP-binding cassette, sub-family C (CFTR/MRP), member 3
KRAS: Kirsten rats arcomaviral oncogene homolog
ALK: anaplastic lymphoma kinase
PD-1: programmed cell death protein 1
c-MYC: Protooncogene
CSF-1: Macrophage Colony Stimulating Factor 1
miRNAs: MicroRNAs
EGR1: Early Growth Response Protein 1
METTL3: methyltransferase 3
SMAD7: drosophila mothers against decapentaplegic protein 7
MFAP2: Microfibrillar-associated protein 2
CLK3: CDC-like splicing factor kinase 3
CNVs: Copy Number Variation
WM: writer modification
HCC: Hepatocellular carcinoma
PPARδ: peroxisome proliferator-activated receptor
CSCs: tumor stem cell
GBMs: glioblastomas
UPR: unfolded protein response
NOX: NADH oxidase
ROS: reactive oxygen species
RMS: RNA modification “writers” score
